# Influence of crop rotation, tillage and fertilization on chemical and spectroscopic characteristics of humic acids

**DOI:** 10.1371/journal.pone.0219099

**Published:** 2019-06-27

**Authors:** Francesco De Mastro, Claudio Cocozza, Andreina Traversa, Davide Savy, Hamada M. Abdelrahman, Gennaro Brunetti

**Affiliations:** 1 Dipartimento di Scienze del Suolo, della Pianta e degli Alimenti, University of Bari, Bari, Italy; 2 Centro Interdipartimentale di Ricerca sulla Risonanza Magnetica Nucleare, University of Naples Federico II, Portici, Italy; 3 Soil Science Department, Faculty of Agriculture, Cairo University, Giza, Egypt; Gifu University, JAPAN

## Abstract

The changes in soil organic matter composition induced by anthropogenic factors is a topic of great interest for the soil scientists. The objective of this work was to identify possible structural changes in humic molecules caused by a 2-year rotation of durum wheat with faba bean, lasted for a decade, and conducted with different agricultural practices in a Mediterranean soil. Humic acids (HA) were extracted at three depths (0–30, 30–60 and 60–90 cm) from a Mediterranean soil subjected to different tillage (no tillage, minimum tillage and conventional tillage), crops (faba bean and wheat), and fertilization. The changes in HA quality were assessed by several chemical (ash, yield and elemental analysis) and spectroscopic techniques (solid-state 13C nuclear magnetic resonance, Fourier transform infrared and fluorescence). The results suggest that the different agronomic practices strongly affected the quality of HA. Smaller but more aromatic molecules were observed with depth, while the fertilization induced the formation of simpler and less aromatic molecules due to the enhanced decomposition processes. Under no tillage, more stable humic molecules were observed due to the less soil aeration, while under conventional tillage larger and more aromatic molecules were obtained. Compared to wheat, more aromatic and more oxidized but less complex molecules were observed after faba bean crop. The inorganic fertilization accelerates the decomposition of organic substances rather than their stabilization. At the end of each crop cycle, humic matter of different quality was isolated and this confirms the importance of the rotation practice to guarantee a diversification of the soil organic matter with time. Finally, no tillage induces the formation of more stable humic matter.

## Introduction

Soil is considered the most important compartment in the carbon cycle. It behaves as a CO_2_-producing system because of the respiration and mineralization of organic matter (OM). In contrast, soils are systems that reduce CO_2_ emissions by means of the fixation and immobilization of organic carbon (OC) in relatively stable forms difficult to decompose, especially through humification processes [1]. Humic substances are supramolecular associations of several heterogenous and relatively small molecules deriving from the degradation and decomposition of biological material. Humic acids (HA) are components of humic substances that appear to be held together mainly by van der Waals interactions, hydrogen bonds, hydrophobic interactions and metal-bridged intermolecular electrostatic bonds [2]. Since HA in themselves are not biologically refractory, they survive in soil due to the strong interactions with minerals, and if the molecules complexed have hydrophobic domains exposed to the soil solution, they can act as condensation nuclei for the supramolecular association. The HA nearest the mineral surfaces are protected from the microbial community, while the external ones would be susceptible to desorption and to microbial attack [3]. Therefore, the larger the hydrophobicity of the biomolecules (long-chain alkyl or aromatic compounds), the slower is their degradation in the soil solution [2, 4].

Stable humic molecules are subjected to lower rate of mineralization and, therefore, the corresponding soils release less CO_2_ [5]. The same humic molecules sequester further carbon in the soil, reducing the negative consequences of climate change in terms of CO_2_ production [6]. Humic acids improve soil health and quality through the amelioration of its physical, biological, and chemical properties [7]. In particular, they constitute an important source of nutrients, promote a good soil structure improving the water retention [8–10], and have the capacity to chemically and physically interact with other components in the environment [11–13].

Most agricultural soils in the Mediterranean area contain small amounts of OM, which is a limiting factor for crops growth and production. Different agronomic practices, such as tillage, inorganic fertilization and crop rotation, influence the biological, chemical and physical properties of soil that in turn, determine changes in the quality of OM from which they originate [14–18]. For example, Moussadek et al. demonstrated that the no tillage, without removing the crop residues from the field, increased the soil organic matter content compared to the conventional tillage [7]. Zhang et al. found HA less complex and less aromatic after the application of the inorganic fertilizers [19]. In a perspective of sustainable agriculture, especially in the Mediterranean area, it is necessary to know the effects of different agronomic practices on the quantity and quality of the soil OM.

A detailed molecular characterization of HA appears, thus, as an essential requirement for evaluating their stability and quality and understanding the role of the different agronomic practices in agricultural and environmental processes. Non-destructive spectroscopic methods such as Fourier transform infrared spectroscopy (FT IR) and ^13^C cross-polarization magic-angle-spinning nuclear magnetic resonance (^13^C-CPMAS-NMR) have been widely used to identify the content and distribution of organic molecules in a wide range of solid organic matrices [20–22]. Fluorescence spectroscopy is another sensitive, non-destructive technique for the evaluation of molecular and quantitative aspects of the structural and functional chemistry of humic substances. The intrinsic fluorescence of soil organic components contains information related to structural components, functional groups, conformation and heterogeneity [23].

Little has been published on the changes in the structure and element composition of HA as affected by tillage, fertilization and crop rotation. The objective of this work is to identify possible structural changes caused by soil management in HA from a Mediterranean soil under different agronomic practices making use of spectroscopic techniques. The results provided could suggest the best agronomic practices in order to conserve the soil organic matter with a view to agronomic and environmental sustainability.

## Materials and methods

### 2.1 Humic acids

The trial was conducted at the experimental station of the University of Bari located at Policoro, latitude 40°10′20″ North and longitude 16°39′04″ East. The experiments included a 2-year rainfed rotation of faba bean (*Vicia faba* Pers. var. equina) cv PROTHABAT 69 with durum wheat (*Triticum turgidum* L. var durum) cv IRIDE, in a split-plot block design with three replications per treatment.

Three different tillage were compared for each crop: (i) conventional tillage (CT, 35 cm deep moldboard plowing in late August and 15 cm deep disk harrowing in November); (ii) minimum tillage (MT, 20 cm deep subsoiling in late August and 15 cm deep disk harrowing in November); and (iii) no tillage (NT).

Regardless the tillage, each new wheat crop received 90 kg N ha^-1^ and each new faba bean crop received 30 kg P_2_O_5_ ha^-1^. Control plots without fertilization were prepared too. Details of the fertilization are reported elsewhere [24].

After a decade of the aforementioned management, soil was sampled from all plots of the faba bean—wheat rotation 2015–2016 at three different depths (0–30, 30–60 and 60–90 cm), after the harvest of each crop. The humic acids (HA) were isolated from: i) NT and no fertilization; ii) NT and crops fertilization; iii) MT and no fertilization; (iv) MT and crops fertilization; (v) CT and no fertilization; (vi) CT and crops fertilization, according to a slightly modified procedure by Swift [25] and reported by Zaccone et al [26].

### 2.2 Chemical characterization of humic acids

Moisture and ash contents were measured by heating the HA for one night at 105°C and for 5 h at 550°C, respectively. The elemental composition was determined by a CHNS Elemental Analyser Flash 2000 (Thermo Scientific) calibrated by a BBOT [2,5-bis-(5- tert.-butyl-benzoxazol-2-yl)-thiophene] standard (ThermoQuest Italia s.p.a.) [27]. Oxygen was calculated by difference: O g kg^-1^ = 1000 **−** (C+H+N+S) g kg^-1^. Data obtained were corrected for moisture and ash contents.

### 2.3 Spectroscopic characterization of humic acids

#### 2.3.1 The E_4_/E_6_ ratio

The E_4_/E_6_ ratio was calculated as the ratio of the absorbances at 465 and 665 nm measured by a Spectrophotometer Perkin Elmer model Lambda 15 UV–Vis on solutions of 3.0 mg of each HA dissolved in 10 ml of 0.05 M NaHCO_3_.

#### 2.3.2 The Fourier transform infrared spectroscopy

The Fourier transform infrared (FT IR) spectra were recorded in the range 4000–400 cm^-1^ on pellets obtained by pressing under reduced pressure a mixture of 1 mg of HA and 400 mg of dried KBr, spectrometry grade, using a Nicolet Nexus FT IR spectrophotometer and a Nicolet Omnic 6.0 software. Spectra were acquired at 2 cm^-1^ resolution, and 64 scans min^-1^ were averaged to reduce noise.

#### 2.3.3 Fluorescence spectroscopy

Fluorescence spectra were obtained on aqueous solutions of HA samples at a concentration of 100 mgl^−1^ after overnight equilibration at RT and adjustment to pH 8 with 0.05 M NaOH [28]. Spectra were recorded using a Perkin Elmer LS 55 luminescence spectrophotometer equipped with the WinLab 4.00.02 software (Perkin–Elmer, Inc., 2001, Norwalk, CT) for data processing. Total luminescence spectra, in the form of excitation–emission matrices (EEMs, contour maps), were recorded over the emission wavelength range from 300 to 600 nm by increasing sequentially by 5 mm step the excitation wavelength from 250 to 500 nm. A scan speed of 1200 nm min^-1^ was selected for both monochromators. The EEM plots were generated as contour maps from spectral data by using Surfer 8.0 software (Golden Software, Inc., 2002, Golden, CO).

The humification index (HIX) was calculated according to Ohno [29]. This index is expressed as the ratio between the area in the upper quarter (435–480 nm) and the sum of the area in the lower quarter (300–345 nm) and in the upper quarter of the emission spectra of HA measured at an excitation wavelength fixed at 254 nm.

#### 2.3.4 NMR Spectroscopy

The ^13^C-CPMAS-NMR experiments were performed on a Bruker AMX 400 operating at 100.625 MHz on the carbon-13. The rotor spin rate was set at 4500 Hz. A recycle time of 2 s and an acquisition time of 13 ms were used. All experiments were conducted with Variable Contact Time (VCT) pulse sequence in order to find the Optimum Contact Time (OCT) for each sample and minimize errors on evaluation of peak areas [30]. The OCT ranged between 0.8 and 1.0 ms. A 50-Hz line broadening was used, and side bands of the carboxyl-C signal were subtracted from the 110–140 ppm region by automatic integration after spectra acquisition [22].

### 2.4 Statistical analysis

All analyses performed on HA were conducted in triplicate. Data were analysed using the four-way ANOVA and Tukey’s test. All statistical analyses were executed using the R software version 3.2.3.

## Results and discussion

### 3.1 Chemical properties of HA

Table 1 shows the effect of the variables depth, tillage, fertilization, and crop (year) on chemical properties of HA. Nitrogen and H content were slightly higher under NT management with respect to the other treatments, in accordance to results by Szajdak et al. [31]. Humic acids incorporate ammonium-N either abiotically, in form of amide-N, or microbiologically, in the form of free or ionized NH_2_-groups in amino acids and sugars, and NH_4_^+^ [32]. The enhanced activity of nitrifiers in more aerated MT and CT plots resulted in a possible major leaching and lower incorporation of N in the corresponding HA.

**Table 1:** Analysis of variance and mean values of the chemical parameters of HA, subdivided by depth, tillage, fertilization and year.

	Ash	N	C	H	S	O	C/N	H/C	O/C	O/H	HA yield
	%	g kg^-1^									g kg^-1^
Depth	n.s.	n.s.	n.s.	n.s.	n.s.	n.s.	n.s.	n.s.	n.s.	n.s.	***
Tillage	n.s.	**	n.s.	*	n.s.	n.s.	*	n.s.	n.s.	*	*
Fertilization	n.s.	*	n.s.	n.s.	n.s.	n.s.	**	n.s.	n.s.	n.s.	n.s.
Crop (year)	n.s.	n.s.	n.s.	n.s.	n.s.	n.s.	n.s.	n.s.	n.s.	n.s.	*
**Depth**											
0–30	5.6 a (0.34)	47.4 a (0.83)	568.2 a (2.37)	50.1 a (0.42)	19.6 a (3.87)	314.6 a (4.74)	14.0 a (0.30)	1.06 a (0.01)	0.42 a (0.01)	0.39 a (0.01)	2.30 c (0.12)
30–60	6.2 a (0.40)	46.5 a (1.53)	577.5 a (3.98)	51.6 a (1.09)	12.1 a (2.28)	312.1 a (5.50)	14.6 a (0.50)	1.07 a (0.02)	0.41 a (0.01)	0.38 a (0.01)	1.36 b (0.17)
60–90	6.2 a (0.35)	47.7 a (1.27)	574.4 a (6.61)	52.5 a (0.78)	23.4 a (6.27)	301.9 a (6.43)	14.2 a (0.52)	1.10 a (0.02)	0.40 a (0.01)	0.36 a (0.01)	0.30 a (0.07)
**Tillage**											
NT	6.1 a (0.42)	49.8 b (1.08)	576.0 a (4.85)	53.1 b (1.07)	21.2 a (6.57)	299.7 a (7.25)	13.6 a (0.32)	1.11 a (0.02)	0.39 a (0.01)	0.35 a (0.01)	1.24 a (0.30)
MT	5.6 a (0.36)	46.8 ab (0.98)	565.1 a (3.21)	50.2 a (0.63)	21.2 a (2.90)	316.6 a (2.85)	14.1 ab (0.38)	1.07 a (0.02)	0.42 a (0.01)	0.39 b (0.01)	1.19 a (0.27)
CT	6.2 a (0.31)	45.0 a (1.23)	579.0 a (5.07)	50.9 ab (0.54)	12.7 a (3.10)	312.3 a (5.25)	15.1 b (0.51)	1.06 a (0.01)	0.41 a (0.01)	0.38 ab (0.01)	1.52 b (0.25)
**Fertilization**											
No	6.2 a (0.30)	45.7 a (1.21)	576.6 a (3.71)	51.0 a (0.76)	15.3 a (2.34)	311.2 a (3.96)	14.9 b (0.45)	1.06 a (0.02)	0.41 a (0.01)	0.38 a (0.01)	1.31 a (0.20)
Yes	5.8 a (0.30)	48.7 b (0.55)	570.1 a (3.85)	51.8 a (0.60)	21.4 a (4.66)	307.9 a (5.30)	13.7 a (0.15)	1.09 a (0.01)	0.41 a (0.01)	0.37 a (0.01)	1.33 a (0.24)
**Crop (year)**											
Faba bean (2015)	5.9 a (0.29)	48.0 a (1.08)	574.2 a (4.39)	51.5 a (0.85)	14.2 a (4.41)	312.1 a (6.02)	14.1 a (0.38)	1.08 a (0.01)	0.41 a (0.01)	0.38 a (0.01)	1.44 a (0.23)
Wheat (2016)	6.1 a (0.31)	46.4 a (0.89)	572.5 a (3.24)	51.3 a (0.49)	22.6 a (2.58)	307.1 a (2.67)	14.5 a (0.34)	1.08 a (0.01)	0.40 a (0.01)	0.37 a (0.005)	1.20 a (0.21)

The values in each column followed by a different letter are significantly different according to Tukey’s test.

* Significant at the P ≤ 0.05

** Significant at the P ≤ 0.01;

*** Significant at the P ≤ 0.001;

n.s.: not significant. The standard errors are reported in parentheses.

No significant difference was observed along the profile and between the two years considered, while the percentage of N was expectedly larger in fertilized soils. The behaviour of N influenced the C/N atomic ratio that was significantly lower in NT and fertilized plots. The atomic ratios H/C and O/C, considered indicators of origin and structural changes of humic substances, did not change significantly in all treatments due to the same origin of HA [33–34]. Further, the smaller O/H ratio of the HA isolated from NT would suggest the occurrence of humic molecules with relatively low polarity and high hydrophobicity [35].

The organic matter content decreased with depth [24], therefore the corresponding HA yield resembled the same trend. With respect to the tillage, the HA yield was slightly larger under CT, in contrast with the results of Moussadek et al. [7] that removed the aboveground crop residues only from the CT plots and not from all treatments, as in present experiments. Finally, fertilization and crop did not influence significantly the HA yield.

### 3.2 UV-Vis properties of HA

The effects of depth, tillage, fertilization and crop (year) on UV-Vis properties of HA are reported in Table 2. Significant differences were observed between E_4_/E_6_ ratios in relation to the tillage. In particular, the E_4_/E_6_ ratio of HA from CT was smaller than those of the other treatments, suggesting the presence of larger humic molecules. In fact, Chen et al. demonstrated that the E_4_/E_6_ ratio is governed by the molecular size, where higher values of E_4_/E_6_ ratio are associated with smaller in size humic molecules, whereas lower E_4_/E_6_ ratio indicates the contrary [36]. In addition, the E_4_/E_6_ ratio slightly increased along the profile, implying the occurrence of smaller or lower in size humic molecules isolated from the deeper horizons, and those results are in accordance to the ones by Marinari et al. [37]. Finally, this ratio was slightly larger in soils after fertilization possibly due to the increased microbial activity that accelerated the decomposition of the organic matter forming humic molecules of smaller size.

**Table 2:** Analysis of variance and mean values of the spectroscopic parameters of HA subdivided by depth, tillage, fertilization, and crop.

	E_4_/E_6_	(2920+2850)/1720	(2920+2850)/1620	Fluorophore A	Fluorophore B	HIX
Depth	*	n.s.	*	**	**	**
Tillage	**	n.s.	*	*	*	*
Fertilization	*	n.s.	n.s.	n.s.	n.s.	**
Crop (Year)	n.s.	***	***	*	**	***
Depth						
0–30	6.12 a (0.08)	1.86 a (0.09)	1.64 b (0.10)	113.0 a (4.16)	108.1 a (5.98)	96.3 a (22.47)
30–60	6.19 a (0.12)	1.81 a (0.08)	1.63 b (0.09)	123.3 a (6.35)	117.3 a (6.79)	141.7 b (31.47)
60–90	6.50 b (0.18)	1.77 a (0.09)	1.57 a (0.09)	165.7 b (5.82)	158.0 b (4.04)	122.3 ab (19.35)
Tillage						
NT	6.39 b (0.11)	1.76 a (0.08)	1.56 a (0.10)	135.3 ab (8.66)	127.3 a (8.33)	128.1 b (24.39)
MT	6.44 b (0.16)	1.83 a (0.09)	1.62 b (0.09)	142.8 b (10.02)	133.7 b (9.50)	116.9 a (24.08)
CT	5.98 a (0.09)	1.85 a (0.10)	1.66 b (0.09)	123.8 a (6.45)	122.3 a (7.82)	115.2 a (27.94)
Fertilization						
No	6.14 a (0.14)	1.81 a (0.07)	1.60 a (0.08)	134.1 a (7.25)	129.3 a (6.77)	138.0 b (26.51)
Yes	6.40 b (0.07)	1.82 a (0.07)	1.62 a (0.08)	133.9 a (6.94)	126.3 a (7.18)	102.2 a (10.28)
Crop (Year)						
Faba bean (2015)	6.26 a (0.09)	1.53 a (0.02)	1.31 a (0.01)	120.9 a (6.08)	114.2 a (6.60)	71.4 a (7.81)
Wheat (2016)	6.28 a (0.13)	2.10 b (0.03)	1.91 b (0.03)	147.1 b (6.58)	141.4 b (5.69)	168.8 b (22.49)

The values in each column followed by a different letter are significantly different according to Tukey’s test. n.s.: not significant;

* Significant at the P ≤ 0.05;

** Significant at the P ≤ 0.01;

*** Significant at the P ≤ 0.001. The standard errors are reported in parentheses.

### 3.3 FT IR spectroscopy

**Fig 1:**
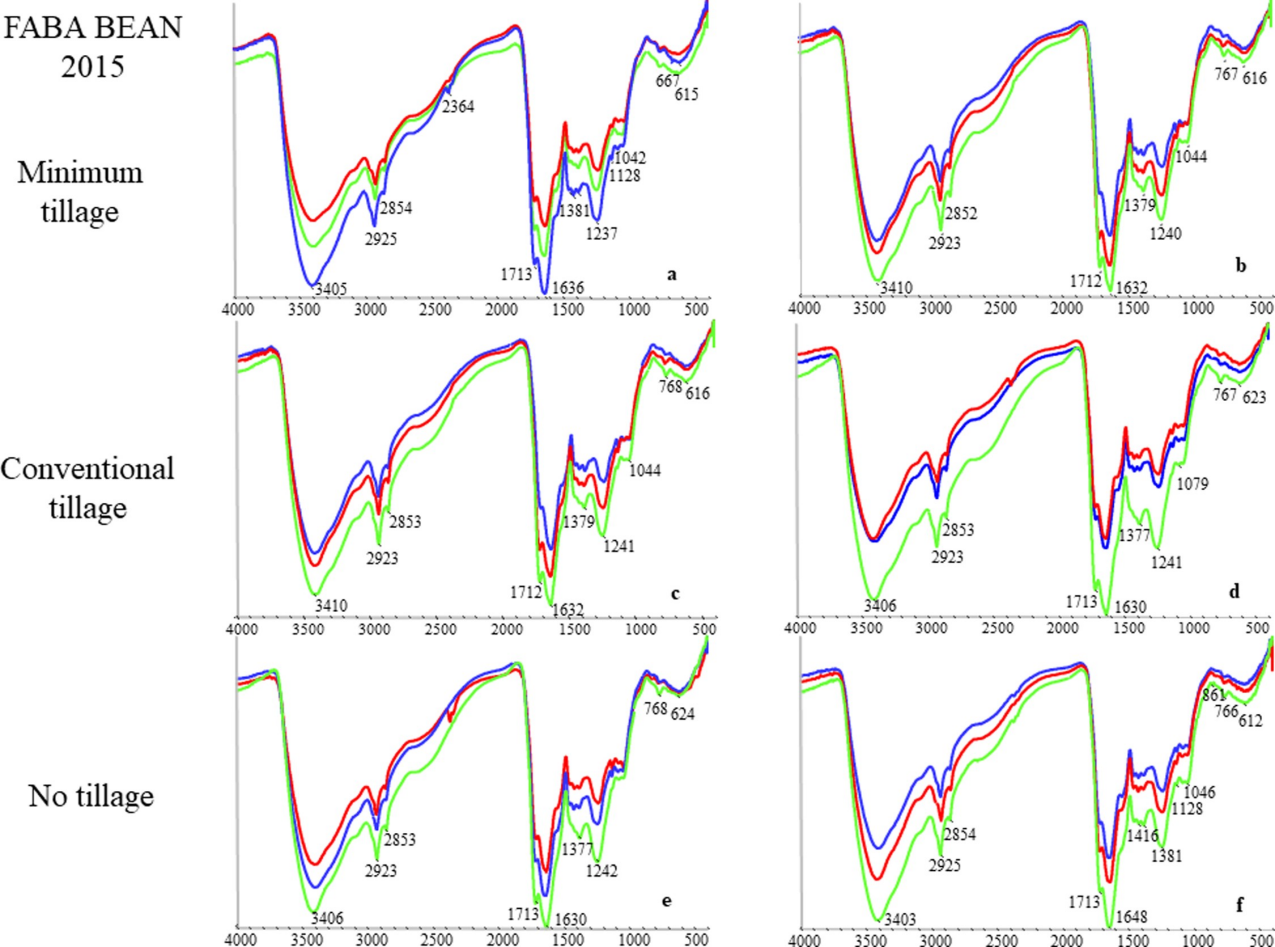
FT-IR spectra of HA samples isolated from faba bean soils, year 2015. a: MT unfertilized; b: MT fertilized; c: CT unfertilized; d: CT fertilized; e: NT unfertilized; f: NT fertilized. Sampling depth: Blu spectra, 0–30 cm; Red spectra, 30–60 cm; Green spectra, 60–90 cm. X-axis: wavenumbers (cm^-1^); Y-axis: transmittance (%).

**Fig 2:**
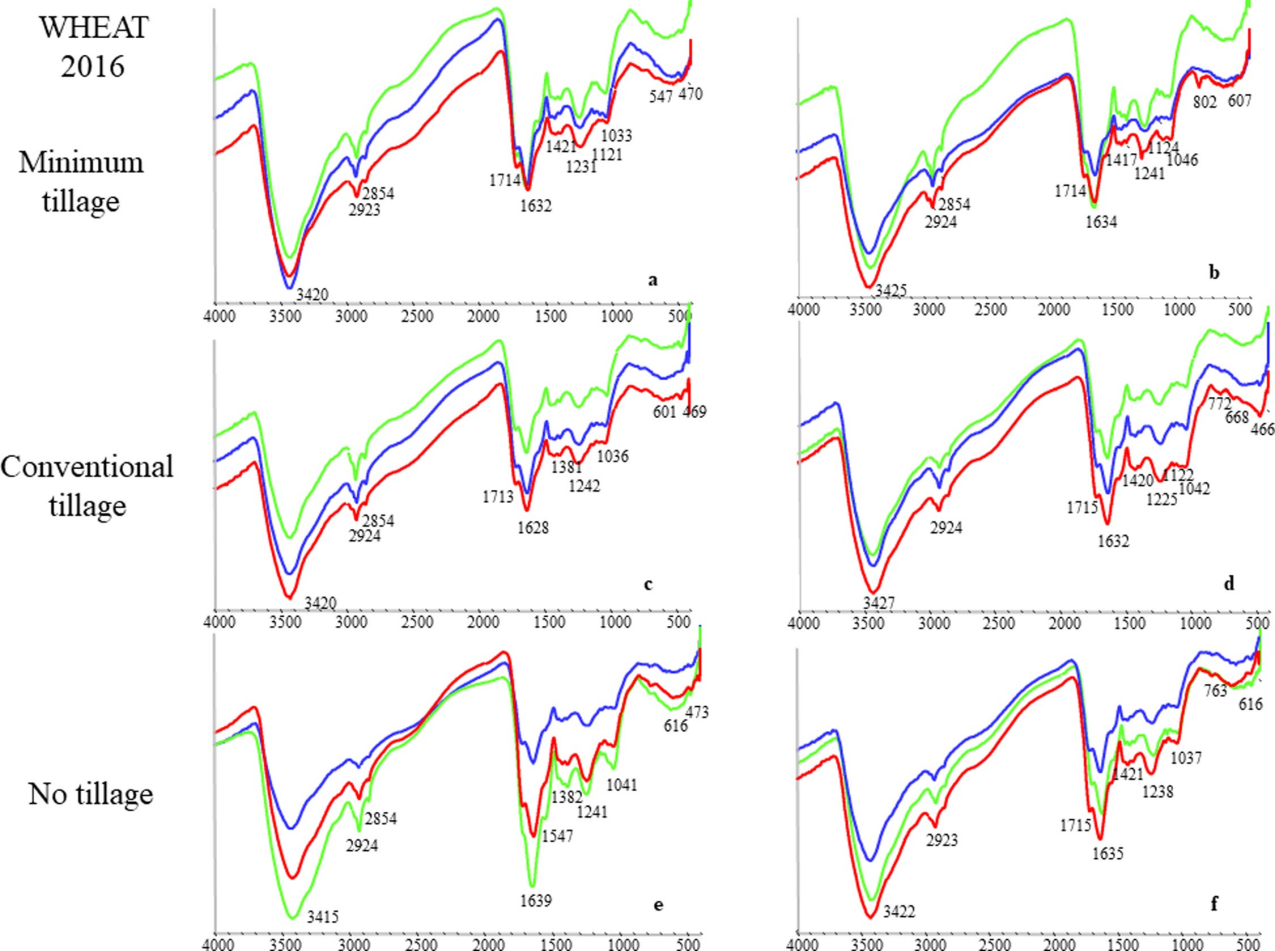
FT-IR spectra of HA samples isolated from wheat soils, year 2016. a: MT unfertilized; b: MT fertilized; c: CT unfertilized; d: CT fertilized; e: NT unfertilized; f: NT fertilized. Sampling depth: Blu spectra, 0–30 cm; Red spectra, 30–60 cm; Green spectra, 60–90 cm. X-axis: wavenumbers (cm^-1^); Y-axis: transmittance (%).

The FT IR spectra of the HA samples isolated in the year 2015 and 2016 under different agronomic management systems are reported in Figs 1 and 2 and showed common absorption bands with some differences in their relative intensity. The most important peaks were: (i) 3400 cm^-1^ associated to OH stretching of OH groups; (ii) 2925 cm^-1^ due to aliphatic C-H stretching; (iii) 1715 cm^-1^ attributed to C = O stretching of COOH and ketones; (iv) 1650 cm^-1^ associated to structural vibrations of aromatic C = C or COO^-^ stretching; (v) approximately 1460 cm^-1^ attributed to asymmetric bending of C-H groups; (vi) approximately 1384 cm^-1^ due to asymmetric stretching of COO^-^ groups, bending of C-H groups, symmetric vibrations of C-NO_2_ groups, vibration of X-N = O groups and asymmetrical stretching of COO^-^ groups and; (vii) 1240 cm^-1^ attributable to C-O stretching and OH bending of COOH groups.

In order to quantify the relative changes in FT IR spectra, the intensity ratios of FT IR peaks 2920+2850 cm^-1^ (aliphatic structures)/1620–1630 cm^-1^ (aromatic structures), and 2920+2850 cm^-1^/1720 cm^-1^ (oxidized structures) of HA were compared according to Zhang et al. (Table 2) [19]. Results suggested a decrease of aliphatic moieties of HA isolated after faba bean, from the NT plots and, generally speaking, with depth.

### 3.4 Fluorescence spectroscopy

**Fig 3:**
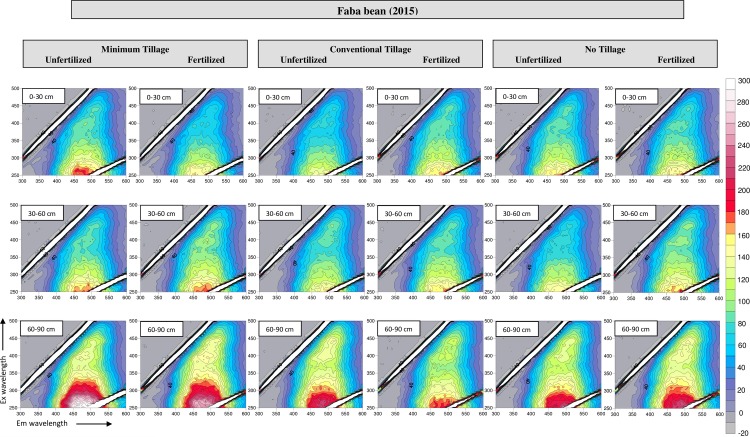
Excitation/Emission matrices of HA samples isolated from faba bean soils, year 2015.

**Fig 4:**
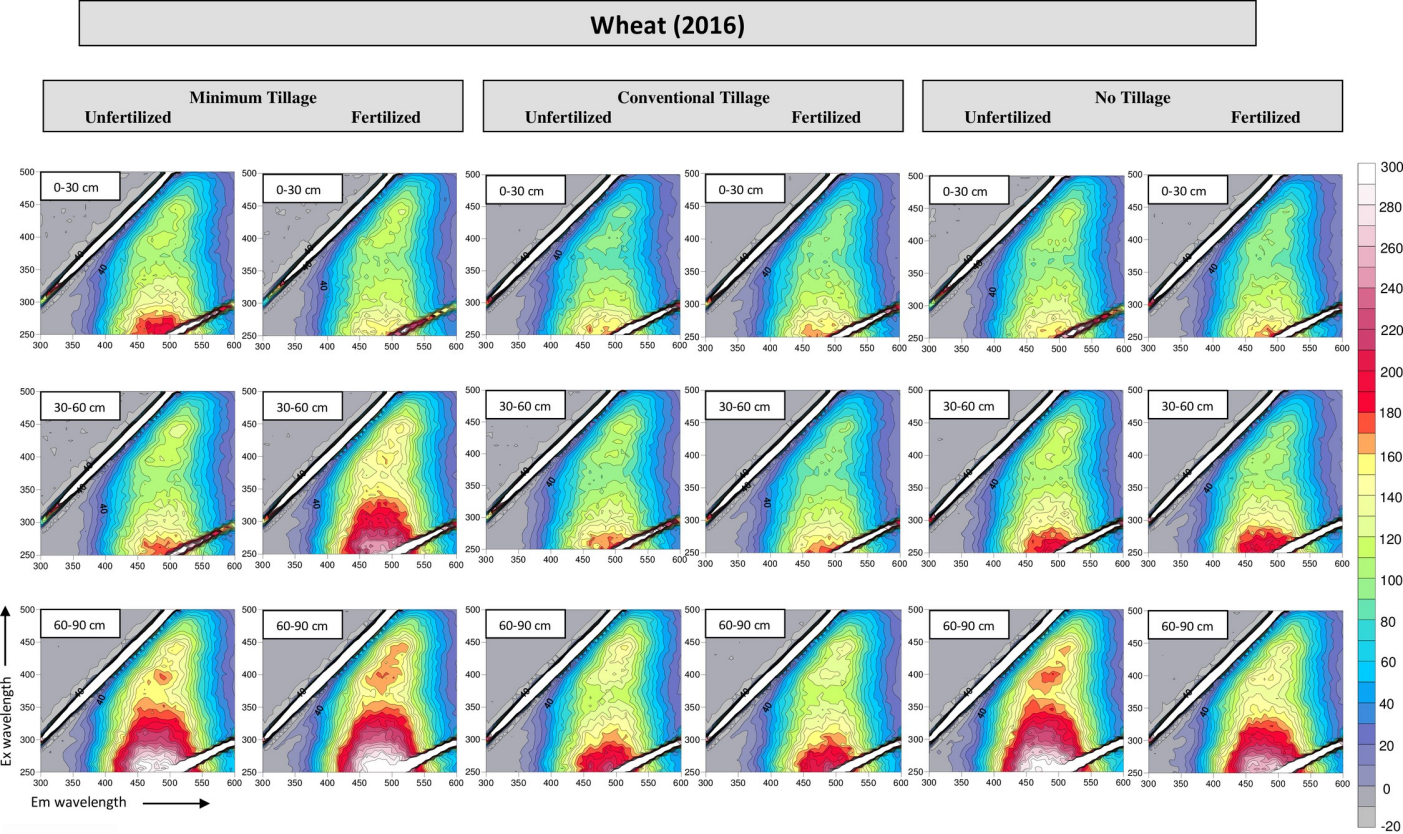
Excitation/Emission matrices of HA samples isolated from wheat soils, year 2016.

The total luminescence spectra of HA samples isolated in the year 2015 and 2016, respectively, are shown in Figs 3 and 4, while in Table 2 are reported the corresponding fluorescence intensity (FI) values of the main fluorophores. The fluorescence investigation revealed the presence of two fluorophores in each sample (A and B), located at an EEWP of about 390ex/480em and 450ex/510em, respectively, ascribed to extensively conjugated quinones and phenols with an elevated polycondensation degree [28]. Miikki et al. associated the FI with the age of HA [38]. In our study, the higher FI observed in the HA isolated after wheat cultivation suggested a more recent formation of the same compared to those found after faba bean cultivation. In addition, the aforementioned higher FI could be related to the presence of substituent groups with strong fluorescence intensity (eg. hydroxys and amidogens) [28]. No evident difference was observed regarding the fertilization, while the FI increasing with depth could be reasonable ascribed to the modification of substituents on the molecule that emitted fluorescence. The lower FI showed in HA under CT could be reasonably ascribed to the higher aromaticity of these HA, as previous confirmed by the (2920+2850)/1620 ratio of the FT IR spectra.

In accord with the results obtained by Tadini et al. [39], the HIX was smallest in the surface horizon (Table 2) possibly due to the presence of fresh debris in the upper layers whose decomposition resulted in the percolation of humic material of low molecular size and high solubility in deeper layers, as reported by Bardy et al. too [40]. This result was in agreement also with Segnini et al. too, who found lower values of humification index in the first layer of soils under different tillage managements, and with the E_4_/E_6_ ratio that suggested the presence of smaller molecules in the deeper layers [41].

The HIX of fertilized soils was smaller than that of the control plots, suggesting an enhanced microbial activity in the former plots that resulted in an increased OM decomposition and in a release of low molecular size humic matter of. Similarly, the different materials produced by the two crops had certainly influenced the HIX with time. In fact, after the faba bean cultivation, a smaller HIX value was found, reasonably attributable to the more suitable C/N ratio of those residues for the microbial community.

### 3.5 ^13^C-CPMAS-NMR spectroscopy

**Fig 5:**
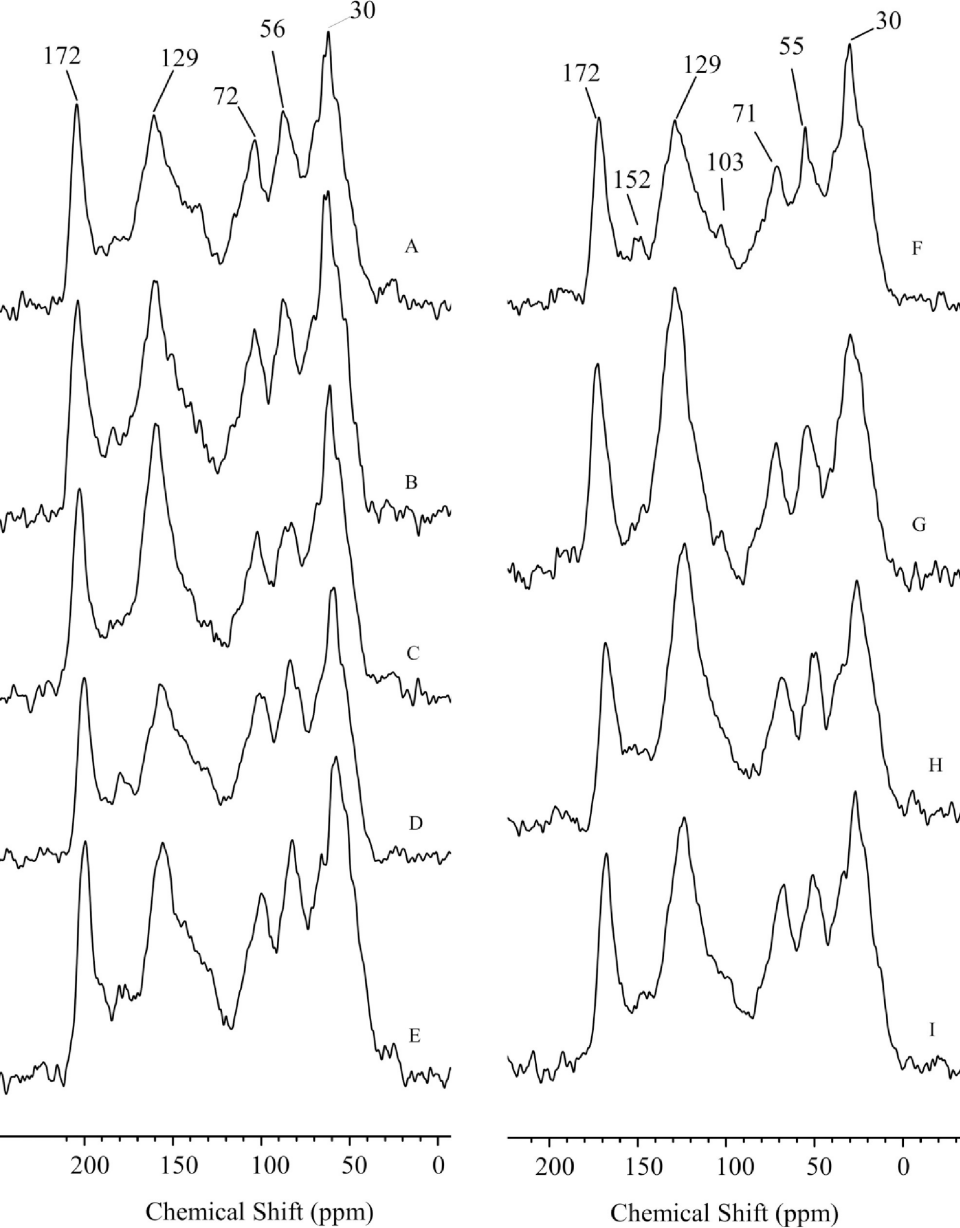
^13^C CP MAS NMR spectra of humic acids isolated from various soil samples. A. NT 2015 0–30 cm; B. NT 2015 30–60 cm; C. NT 2015 60–90 cm; D. CT fertilized 2015 0–30 cm; E. CT 2015 0–30 cm; F. MT 2015 30–60 cm; G. MT 2016 30–60 cm; H. CT 2016 30–60 cm; I. NT 2016 30–60 cm.

The NMR spectra of HA samples are shown in Fig 5, while the corresponding peak assignment and the semi-quantitative results are reported in Table 3. The peaks within the chemical shift region 0–45 ppm have been assigned to alkyl chains, while those between 45 and 60 ppm are usually associated with the resonance of lignin methoxy groups or C-N in amino acids [42]. Moreover, signals in the region 60–110 ppm have been attributed to *O*-Alkyl-containing molecules, mainly carbohydrates, whereas peaks within the 110–160 ppm interval arose from the resonance of aromatic and phenolic carbons, such those in lignin, tannins or resins [43–45]. Finally, signals within the 160–210 ppm range were assigned to the carboxyl and amide functional groups [43].

**Table 3:** Relative carbon distribution (%) for different chemical shift regions (ppm) in 13C-CPMAS-NMR spectra (Fig 5) of HA extracted from soils managed with different tillage systems: No Tillage (NT), Conventional Tillage (CT) and Minimum Tillage (MT). The HA samples have been extracted at different depths: 0–30, 30–60 or 60–90 cm.

Attribution	Chemical shift range (ppm)	NT 0–30 cm 2015	NT 30–60 cm 2015	NT 60–90 cm 2015	CTF 0–30 cm 2015	CT 0–30 cm 2015	MT 30–60 cm 2015	MT 30–60 cm 2016	CT 30–60 cm 2016	NT 30–60 cm 2016
**Alkyl-C**	45–0	24.0 a (0.17)	28.3 b (0.52)	27.8 b (0.58)	29.0 b (0.38)	27.1 a (0.24)	26.8 b (0.14)	23.9 a, A (0.07)	23.1 A (0.32)	26.7 B (0.24)
**Methoxyl-C**	64–45	13.0 b (0.51)	12.7 ab (0.44)	11.7 a (0.31)	13.7 a (0.32)	13.3 a (0.29)	12.6 a (0.32)	11.1 a, A (0.09)	11.3 A (0.03)	12.3 B (0.22)
***O*-Alkyl**	105–64	18.8 b (0.32)	17.1 ab (0.23)	16.3 a (0.21)	17.5 a (0.20)	18.4 a (0.22)	17.6 b (0.09)	16.3 a, A (0.19)	17.5 A (0.12)	18.1 A (0.10)
**Aromatic-C**	145–105	24.0 a (0.40)	23.5 a (0.40)	27.2 b (0.47)	22.3 a (0.31)	23.9 b (0.23)	24.1 a (0.09)	30.4 b, B (0.15)	30.5 B (0.23)	25.9 A (0.19)
**Quaternary aromatic C**	160–145	5.4 a (0.10)	5.0 a (0.21)	4.9 a (0.21)	5.1 a (0.23)	5.0 a (0.20)	4.9 a (0.12)	4.9 a, A (0.23)	5.8 B (0.24)	4.7 A (0.17)
**Carboxyl-C, Amide-C**	210–160	14.7 b (0.32)	13.4 ab (0.25)	12.0 a (0.40)	12.4 a (0.29)	12.3 a (0.25)	14.0 a (0.35)	13.5 a, B (0.37)	11.9 A (0.03)	12.3 A (0.12)
**Alkyl/*O*-Alkyl**		1.3 a (0.02)	1.7 b (0.01)	1.7 b (0.05)	1.7 b (0.03)	1.5 a (0.03)	1.5 a (0.002)	1.5 a, A (0.02)	1.3 A (0.02)	1.5 A (0.01)

All the measurements refer to HA isolated from non-fertilized soils, except for CTF (CT fertilized).

Different letters in a row indicate significant differences according to the Tukey’s range post hoc test (p < 0.05). A One-Way ANOVA was carried out to compare the means of HA from NT soils (2015) at increasing depth, and another one for comparing HA from different tillage systems (MT, CT and NT 2016, uppercase letters). The HA from CTF and CT, as well as those from MT soils after faba bean (2015) and wheat cultivation (2016), were compared by using the t-test (p < 0.05). The standard errors are reported in parentheses.

Along the profile of soils under NT (Fig 5, spectra A, B and C; Table 3), an increase of alkyl- and aromatic/phenolic-related signals in HA can be noted, while the content of methoxyl-C, carbohydrates and carboxyl moieties significantly decreased. An increased amount of aromatic molecules, together with a concomitant decrease of *O*-Alkyl compounds, may indicate a larger stability of HA with soil depth, due to a larger protection of SOM from microbial degradation [45]. Such result is also supported by the significantly larger Alkyl-C to *O*-Alkyl-C ratio found at increasing depths (Table 3). Such parameter is commonly employed as an index for the degradation degree of SOM [46].

The HA isolated from fertilized soils under CT (Fig 5, spectrum D; Table 3) showed a larger relative content of alkyl-C (0–45 ppm) and a reduction of the amount of *O*-Alkyl and aromatic moieties with respect to the HA isolated from unfertilized soils under CT (Fig 5, spectrum E; Table 3). These results agree with those reported by Drosos and Piccolo [18], who found an increased content of alkyl C and a decreased content of aromatic C in HA due to the application of an inorganic fertilizer. According to Galantini and Rosell [47], the fertilization promotes an increase of aliphatic groups in respect to aromatic moieties, probably due to the large inputs in fertilized than in *non*-fertilized soils, thus increasing the amount of less transformed organic compounds, which were bound to HA. Such results are well in line with the E_4_/E_6_ ratio and the HIX showed before.

Humic acids isolated from soils under MT after faba bean (2015; Fig 5, spectrum F and Table 3) contained a significantly larger relative content of alkyl- and *O*-Alkyl-C than HA isolated from soils under MT after wheat (2016; Fig 5, spectrum G and Table 3). Conversely, the relative amount of aromatic/phenolic moieties significantly increased after wheat cultivation (Table 3). A reduced content of Alkyl-C has been associated with an oxidation of SOM, thus suggesting the occurrence of SOM degradation processes [48].

Finally, the comparison among the three tillage systems studied (Fig 5, spectra G, H and I; Table 3) showed a larger relative content of Alkyl-, methoxy- and *O*-Alkyl-C and a lower relative amount of aromatic and phenolic moieties in HA from NT compared with that from CT. Conversely, the content of such functional groups in HA from MT soils was either intermediate or comparable to that found for HA from CT, except for the content of carboxyl groups, which was significantly larger in HA from MT soil than in those from CT soils (Table 3). The general trend observed here is in line with previous reports where large amount of *O*-Alkyl groups in NT were related to a lesser degradation of carbohydrates [49]. This finding, together with the smaller relative content of both carboxyl moieties and Alkyl-C and a larger Alkyl/*O*-Alkyl ratio for soils under conservational tillage practices (Table 3), suggested a lower SOM degradation for these soils [48]. Indeed, the larger stability found for the HA under NT may be due to the fact that, under this tillage system, soils were less disturbed, and the growth of SOM-decomposing microbes was limited, thus inhibiting the rate of SOM degradation [45].

## Conclusions

The present study reports the effects of different soil managements on quality and quantity of HA when the aboveground crop residues have been removed from all treatments. The NT apparently induced the formation of HA more stable that can contribute to the soil fertility for a longer time. In contrast, CT induced the formation of a slightly greater quantity of HA with more aromatic and larger structure. The inorganic fertilization, favoring a more intense OM decomposition, induced the formation of simpler and more aliphatic humic molecules that should persist less time in the soil. Humic acids extracted at the end of the legume crop showed more aromatic and oxidized character but less complex structure with respect to the ones isolated after the wheat crop. Finally, smaller but more aromatic HA were observed with depth.

Future investigations should interest the impact of the variables studied (tillage, fertilization, crop and depth) on HA when the aboveground crop residues return to soil. In that case, one can predict a major yield of HA, that influence positively the soil fertility, but the effects on the HA quality could differ from the ones of the present study.

## Supporting information

S1 Dataset Results of chemical and spectroscopic analyses. (XLSX)
